# Making National Cancer Institute–Designated Comprehensive Cancer Center Knowledge Accessible to Community Oncologists via an Online Tumor Board: Longitudinal Observational Study

**DOI:** 10.2196/33859

**Published:** 2022-05-19

**Authors:** Maitri Kalra, Elizabeth Henry, Kelly McCann, Meghan S Karuturi, Jean G Bustamante Alvarez, Amanda Parkes, Robert Wesolowski, Mei Wei, Sarah S Mougalian, Gregory Durm, Angel Qin, Caitlin Schonewolf, Meghna Trivedi, Avan J Armaghani, Frederick H Wilson, Wade T Iams, Anita A Turk, Praveen Vikas, Michael Cecchini, Sam Lubner, Priyadarshini Pathak, Kristen Spencer, Vadim S Koshkin, Matthew K Labriola, Catherine H Marshall, Katy E Beckermann, theMednet.org NCI-CCC Tumor Board Program Collaborative Group, Marina N Sharifi, Anthony C Bejjani, Varsha Hotchandani, Samir Housri, Nadine Housri

**Affiliations:** 1 Division of Hematology/Oncology Department of Medicine Indiana University Health Ball Memorial Hospital Fishers, IN United States; 2 Division of Hematology/Oncology Department of Medicine Loyola University Stritch School of Medicine Maywood, IL United States; 3 Division of Hematology/Oncology Department of Medicine University of California, Los Angeles Beverly Hills, CA United States; 4 Division of Hematology/Oncology Department of Medicine The University of Texas MD Anderson Cancer Center Houston, TX United States; 5 Division of Hematology/Oncology Department of Medicine West Virginia University Morgantown, WV United States; 6 Division of Hematology/Oncology Department of Medicine University of Wisconsin Madison, WI United States; 7 Division of Hematology/Oncology Department of Medicine Ohio State University Columbus, OH United States; 8 Division of Hematology/Oncology Department of Medicine University of Utah Utah City, UT United States; 9 Department of Radiation/Oncology Yale University School of Medicine New Haven, CT United States; 10 Division of Hematology/Oncology Department of Medicine Indiana University School of Medicine Indianapolis, IN United States; 11 Department of Radiation Oncology University of Michigan Ann Arbor, MI United States; 12 Division of Hematology/Oncology Department of Medicine, Herbert-Irving Comprehensive Cancer Center Columbia University New York, NY United States; 13 Division of Hematology/Oncology Department of Medicine Moffitt Cancer Center University of South Florida Tampa, FL United States; 14 Division of Oncology Department of Medicine Vanderbilt University Nashville, TN United States; 15 Division of Hematology/Oncology Department of Medicine University of Iowa Iowa City, IA United States; 16 Division of Hematology/Oncology Department of Medicine Rutgers University Cancer Institute of New Jersey New Brunswick, NJ United States; 17 Division of Hematology/Oncology Department of Medicine Duke University Durham, NC United States; 18 Division of Oncology Department of Medicine Johns Hopkins University Baltimore, MD United States; 19 Mednet Inc New York, NY United States; 20 Division of Hematology/Oncology Department of Medicine Veterans Health Administration Greater Los Angeles Health System Los Angeles, CA United States

**Keywords:** National Cancer Institute–designated Comprehensive Cancer Centers, NCI-CCC, tumor boards, TBs, knowledge sharing, cancer, digital health, oncology, health websites, health education

## Abstract

**Background:**

Expert knowledge is often shared among multidisciplinary academic teams at tumor boards (TBs) across the country, but these conversations exist in silos and do not reach the wider oncology community.

**Objective:**

Using an oncologist-only question and answer (Q&A) website, we sought to document expert insights from TBs at National Cancer Institute–designated Comprehensive Cancer Centers (NCI-CCCs) to provide educational benefits to the oncology community.

**Methods:**

We designed a process with the NCI-CCCs to document and share discussions from the TBs focused on areas of practice variation on theMednet, an interactive Q&A website of over 13,000 US oncologists. The faculty translated the TB discussions into concise, non–case-based Q&As on theMednet. Answers were peer reviewed and disseminated in email newsletters to registered oncologists. Reach and engagement were measured. Following each Q&A, a survey question asked how the TB Q&As impacted the readers’ practice.

**Results:**

A total of 23 breast, thoracic, gastrointestinal, and genitourinary programs from 16 NCI-CCC sites participated. Between December 2016 and July 2021, the faculty highlighted 368 questions from their TBs. Q&As were viewed 147,661 times by 7381 oncologists at 3515 institutions from all 50 states. A total of 277 (75%) Q&As were viewed every month. Of the 1063 responses to a survey question on how the Q&A affected clinicians’ practices, 646 (61%) reported that it confirmed their current practice, 163 (20%) indicated that a Q&A would change their future practice, and 214 (15%) reported learning something new.

**Conclusions:**

Through an online Q&A platform, academics at the NCI-CCCs share knowledge outside the walls of academia with oncologists across the United States. Access to up-to-date expert knowledge can reassure clinicians’ practices, significantly impact patient care in community practices, and be a source of new knowledge and education.

## Introduction

Cancer is the second leading cause of death in the United States [1]. The field of oncology is rapidly evolving, and it is difficult to stay up-to-date with the changing treatment paradigms. In 2017, the Food and Drug Administration (FDA) issued 58 new approval notifications in hematology/oncology—more than in any other field of medicine [2]. In 2019, 13 of the 48 novel drugs approved by the FDA were in the field of hematology/oncology [3]. As a result of the rapidly changing treatment practices, clinicians often have questions regarding the management of specific clinical scenarios [4]. However, clinical trials and clinical practice guidelines often do not answer questions on complex and nuanced clinical situations [5-7]. When clinicians search current resources and do not find an answer, they are often faced with having to make difficult clinical judgments without sufficient expertise in the particular clinical scenario [6]. Therefore, more than half of the questions go unanswered, which may result in inconsistent and poor quality of patient care [4-7]. Additionally, there is a lost opportunity for knowledge gaps to be identified and targeted.

In oncology, difficult clinical scenarios are often discussed within a multidisciplinary tumor board (TB). The TBs at the National Cancer Institute–designated Comprehensive Cancer Centers (NCI-CCCs) serve as excellent opportunities for experts to share their knowledge. These discussions can play a crucial role in impacting patient care and survival [8,9]. Unfortunately, the TB insights from experts at the NCI-CCCs are not systematically documented and disseminated in a way that is easily accessible to physicians in the community. This represents a lost opportunity to capture and share real-world questions, thoughtful discussions, and clinical expertise that can impact patient care in community centers. This paradigm can change using social networks, which have long been acknowledged as critical for the diffusion and adaptation of new information and experiential physician knowledge.

In other industries, social question and answer (Q&A) databases have become a method of knowledge creation and storage, which can be ranked via a search engine and discovered by all internet users. The most well-known examples of such databases are Stack Overflow and Quora, which owe their success to having significant user bases with deep expertise in their domains [10]. Building on the utility of Q&A databases, theMednet was developed in 2014 as a physician-only online platform with a mission to facilitate knowledge sharing from academic to community physicians in order for patients to get high-quality care despite where they are treated. It was designed for community oncologists to ask non–case-based clinical questions from experts and for the expert answers to be part of a large and searchable Q&A database that would be accessible at any time to physicians with similar questions. In effect, this would bring the Q&A process in medicine to an online platform and expose community clinicians to strong expert networks. It was started among radiation oncologists across the United States and then expanded to involve medical, surgical, and pediatric oncologists. Experts and community oncologists join theMednet through individual outreach, invitations from users, and word of mouth. All members are reviewed to ensure that they are US-based practicing oncologists. The platform is moderated by a team of deputy and associate editors who review every question, answer, and comment posted. TheMednet now contains over 10,000 clinical questions that cannot be easily answered based on a review of the literature, textbooks, or guidelines. Over 13,000 US radiation oncologists, medical oncologists, surgical oncologists, gynecologic oncologists, and pediatric oncologists are registered members, with 50% of registered physicians using the website at least once a month.

Having both an established community of academic and community oncologists and a content management system that routes questions to the appropriate experts and routes answers for appropriate peer review, theMednet is uniquely suited to capture and disseminate knowledge from the NCI-CCCs to community oncologists. We sought to use theMednet to document, discuss, and disseminate clinical knowledge from the TBs at the NCI-CCCs to the oncology community, using technology and best practices from online social networks.

## Methods

### Overview

We hypothesized that the experts at the NCI-CCCs can systematically document and share experiential knowledge and best practices into actionable information in the form of searchable Q&As on theMednet. We also hypothesized that the Q&As from the TBs, in addition to the clinical Q&As from the community, have a long-lasting value to future users who will have similar questions, which may have otherwise gone unanswered. While the term “expert” may have many connotations and definitions, in the context of this application we define “expert” as an oncology specialist (medical, radiation, surgical, etc) with an academic appointment at a US university, who has published original research in his or her subspecialty (eg, breast cancer, prostate cancer, etc) or participates in clinical trials related to that specialty.

**Figure 1:**
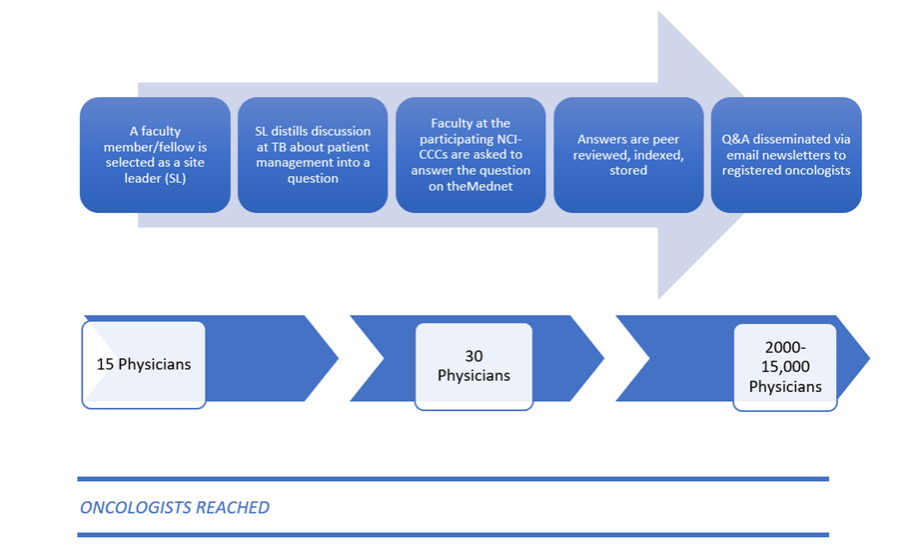
Methodology and potential reach. NCI-CCCs: National Cancer Institute–designated Cancer Centers; Q&A: question and answer, TB: tumor board.

The program was initiated at a single site as a pilot program with the University of Texas MD Anderson Cancer Center (MDACC). In the pilot, we collaborated with the breast cancer faculty at the MDACC, an NCI-CCC treating over 135,000 patients with cancer a year, to jointly develop a process that distills, documents, and distributes important information from the TBs via theMednet. In this process, a junior faculty member was assigned as a “site leader” to post 1 question per week from the TB. The question would be routed to a physician editor who would then invite experts from the NCI-CCC and other academic cancer centers to answer the question. The answers would then be distributed to additional faculty for peer review. By the end of the week, the Q&A would be included in a weekly email newsletter and distributed to the physician members of theMednet. Through this process, a discussion among 15 to 20 physicians at a single time and place becomes part of a searchable repository of knowledge that provides long-lasting value to 500 times more physicians (Figure 1).

Once the program was successfully launched and running at the MDACC, further expansion was focused on breast cancer sites at NCI-CCCs across the country. Institutions designated as NCI-CCCs with a high level of engagement on theMednet were selected. In the next phase, the program was expanded to 5 sites in thoracic oncology, followed by 4 sites in gastrointestinal oncology and 4 sites in genitourinary oncology. At each site, a site leader was selected to distill discussions about patient management from the TB meetings into questions to be posted on theMednet. Experts from medical oncology, radiation oncology, and surgical oncology were invited to participate as experts from each site. Web- or phone-based training sessions for site leaders and expert physicians were held prior to each launch with further details provided below.

### Information Creation and Quality Review

Site leaders were instructed to distill conversations about a patient case into one or more questions to be posted on theMednet. A training manual was provided to each site leader to explain how to write questions in a way that concisely addresses the clinical situation being discussed and not the specific patient case. Writing the questions in this manner facilities search queries and encourages the answer to be written in a way that applies to a broad range of patients, providing guidance and educational value to a greater number of physicians. It also removes any information that would violate the Health Insurance Portability and Accountability Act standards. Additionally, questions were required to be focused on nuanced clinical discussions, where there were no clear answers based on existing research and guidelines. If a similar question already existed in theMednet database, that old question was tagged as the “tumor board question” and then sent to the TB experts for updated answers as responses may have changed based on evolving data.

Once a question was posted on theMednet, it was sent to 3 or 4 physicians participating in the TB program via email to be answered. Answers were then shared with other experts nationwide for peer review. This process either built consensus around a course of action for a clinical situation or created a dialogue around best practices when there was no clear answer. The pool of experts for peer review included academic medical oncologists at the participating NCI-CCCs and academic medical oncologists across the country who had previously been recruited to answer questions from community oncologists. Q&As were reviewed and indexed by physician editors for easy search retrieval.

### Information Dissemination and Ongoing Engagement

A customized email, highlighting new answers from the TB conference, was sent biweekly to oncologists registered on theMednet. Because of the increased volume of the Q&As over time, a daily digest was also created for users who opted to receive a daily, rather than a biweekly, email. The newsletter went out daily to 5617 medical and radiation oncologists. It was sent biweekly to 5018 medical and radiation oncologists. Questions were also included in weekly newsletters to 1277 pediatric and gynecologic oncologists if relevant to those specialists.

To provide feedback to experts answering questions and highlight their impact, a bimonthly custom report was created detailing the number of times their answers were read, the number of physicians their answers reached, and the number and names of the institutions their answers reached. The site leaders received an automated email just before their TB meeting every week to remind them to post a question, with the expectation of posting 1 question a month. A TB project manager individually contacted site leaders at each site at the end of a month if at least 1 question had not been received from them that month.

### Target Audience and Dynamic Feedback

To help actively capture the opinions and real-world practices of oncologists using theMednet, 1-question polls were created for a number of TB questions. Community physicians also provided feedback by marking a question as a “good question” or indicating whether they “agree” with or find answers “helpful.” An additional survey also captured whether the information in the Q&A had changed their practice or confirmed their current practice. Both the total views and the views per unique physicians for each individual Q&A were tracked over time.

### Ethics Approval 

This analysis was exempt from IRB review as it does not include human subjects research and involves secondary analysis of published online data. Impact surveys were issued by site personnel for the intent and purposes of improving services and programs for members. The privacy of users was protected, and confidentiality of individual responses was maintained throughout data collection and review. Results from data analysis are being presented in aggregate.

## Results

### The NCI-CCC Sites

The NCI-CCC breast cancer TB program was initially launched in December 2016 with the MDACC and expanded to include the University of Pittsburg Medical Center and the University of California, Los Angeles by April 2017. Between April 2017 and July 2021, the program was expanded to include a total of 23 breast, thoracic, and gastrointestinal programs at 16 NCI-CCCs indicated in Table 1. A total of 22 out of 23 TB programs were retained at the time of this publication. Only 1 program declined further participation because of the inability of a site leader to participate. The program grew from 38 involved academic physicians in 2017 (6 faculty members asked questions that were answered by 32 experts) to 131 academic physicians by July 2021 (16 faculty members asked questions that were answered by 69 experts.)

**Table 1:** The National Cancer Institute–designated Comprehensive Cancer Center tumor board (TB) participating sites.

TB program	Participating sites
Breast cancer	MD Anderson Cancer Center; University of California, Los Angeles; Yale Cancer Center; University of Utah; University of Wisconsin; Columbia University Medical Center; The Ohio State University Medical Center; Moffitt Cancer Center; University of Iowa
Thoracic malignancies	Indiana University, The Ohio State University Medical Center, Yale Cancer Center, Vanderbilt University Medical Center, University of Michigan
Gastrointestinal malignancies	University of Wisconsin, Yale Cancer Center, Indiana University, Rutgers Health
Genitourinary malignancies	Duke University Medical Center; Vanderbilt University Medical Center; The Sidney Kimmel Comprehensive Cancer Center, Johns Hopkins University; University of California, San Francisco

### Q&A Reach

**Figure 2:**
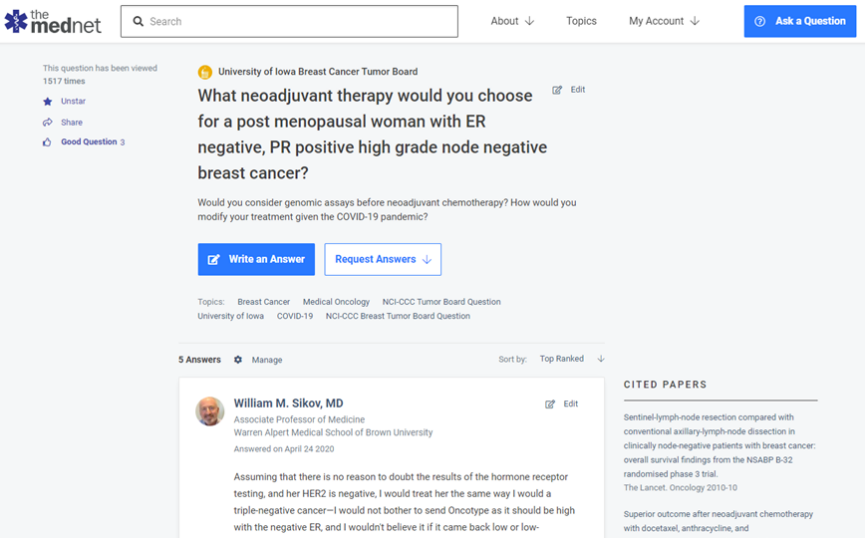
Example of a question asked in the breast cancer tumor board program with an expert response and peer review. ER: estrogen positive; PR: progesterone positive.

**Figure 3:**
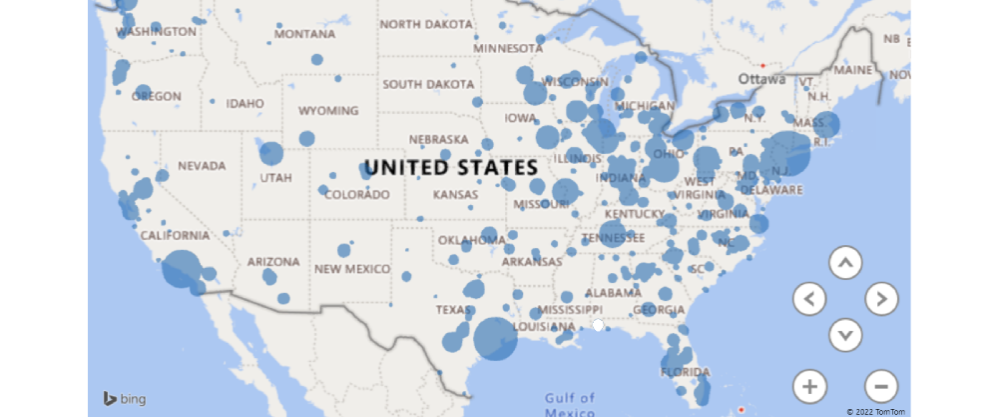
Map of the United States showing the reach of tumor board program.

Between December 2016 and July 2021, a total of 534 answers to 368 questions have been posted from these 23 programs from 16 NCI-CCC sites. Answers came from 123 academic physicians and were peer reviewed by 93 academic physicians. A total of 127 (35%) questions had more than 1 answer. Figure 2 shows a typical format of how a TB question, asked from a site leader, is answered by an expert from a different site and then peer reviewed by another expert in the field. These Q&As were viewed 147,661 times by the oncologists at 3515 institutions from all the 50 states of the United States, including 5131 community oncologists (Figure 3). A total of 227 (75%) Q&As were viewed every month. Answers to 22 questions were updated at least 6 months after the initial answers due to evolving data in the field.

**Figure 4:**
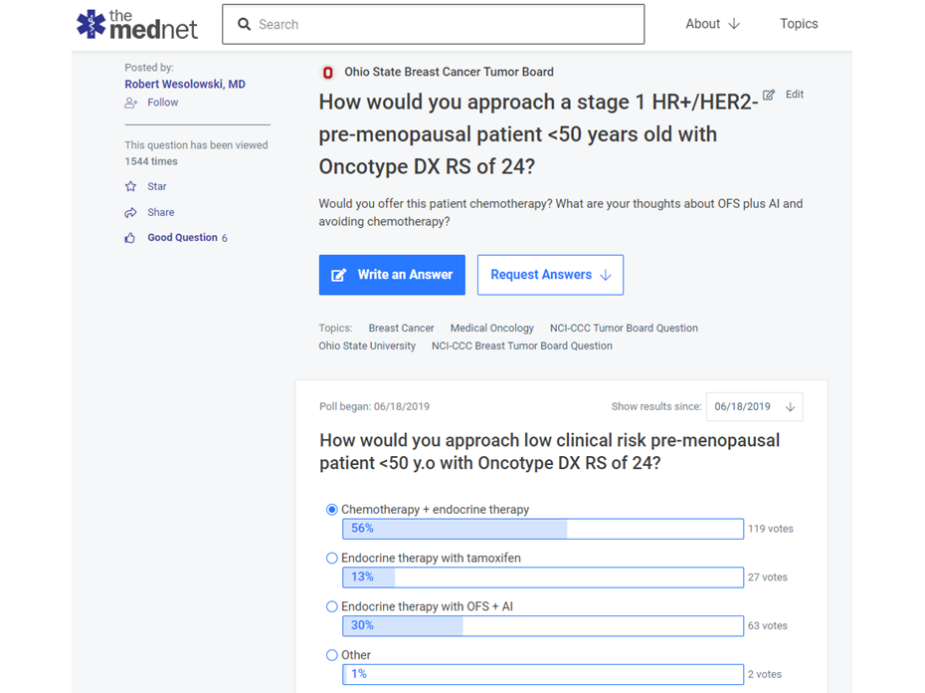
Example of a real-world practice poll question with responses. HER2: human epidermal growth factor receptor 2; HR: hormone receptor; oncotype DX; 21 gene recurrence score; RS: recurrence score.

A total of 431 clinicians agreed with the answers 1773 times, and 545 physicians found them helpful 1321 times. Editors created 88 (24%) real-world practice poll questions out of these Q&As. These poll questions asked for the clinical opinion of the oncologists about a particular scenario (Figure 4). A total of 2116 clinicians voted in these polls with 7789 votes.

A total of 231 (43%) answers cited published data with 328 publications cited. Nearly 303 (60%) of the answers cited clinical experience, highlighting the frequency with which oncologists encounter scenarios not adequately addressed by the current evidence. Customized emails with new Q&As resulted in a visit to the website an average of 15% of the time (7 times the industry average) [11].

### Dynamic Feedback

**Figure 5:**
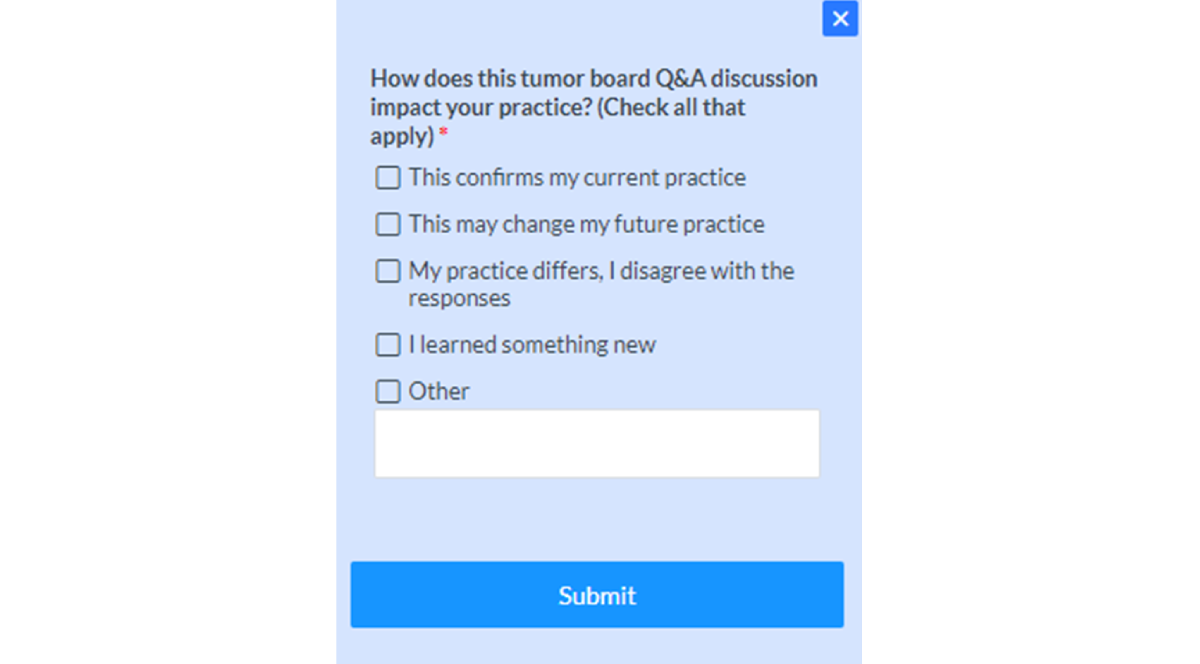
Impact survey questions.

We conducted a short survey on how the Q&As impacted the clinicians’ practice. The survey questions were posted at the end of each Q&A page and were open to response by the viewers. The impact survey questions are listed in Figure 5. Of the 1063 responses to a survey on how the Q&As affected clinicians’ practice, 646 (61%) reported that it confirmed their current practice, 163 (20%) indicated that a Q&A would change their future practice, 214 (15%) reported learning something new, 20 (2%) indicated that their practice differs, and 20 (2%) chose “other” as their response. Multimedia Appendix 1 shows a pie graph of all the impact survey question responses.

Table 2 summarizes the number of views and the number of oncologists engaged over time. We have found that both the Q&A views and the number of oncologists viewing the TB Q&As increased over time. A total of 277 (75%) of all the TB Q&As were viewed every month.

In February 2020, qualitative feedback was sought from 8 site leaders. Standardized questions were developed and focused on process improvement. Sample questions included “Would you recommend this program to other NCI-CCC sites? Why or why not?” and “What are some barriers to posting questions?” Eight out of the 8 site leaders stated they would recommend this program to other NCI-CCCs. Some of the feedback was that the program “opened up conversations at our institution”; “has helped expand my knowledge base”; “helps hearing what is going on at other sites”; and “some answers don’t have the strongest evidence but it is good to know who agrees.” The most common barriers to posting a question routinely were (1) the time to distill a clinical scenario into a broad-based question and (2) not being able to identify a good question from the TB. Of the 8 site leaders, 7 found automated email reminders useful to remember to think of a question to share during the TB discussions.

**Table 2:** Community oncologists’ engagement over time.

Year	Oncologists viewing the TB^a^ Q&As^b^, n	TB Q&A views, n
2016	172	361
2017	844	4792
2018	3010	24,886
2019	3616	39,550
2020	4749	49,941

^a^TB: tumor board.

^b^Q&A: question and answer.

## Discussion

Oncology is a constantly evolving field of medicine, and oncologists deal with complex patients and clinical scenarios every day due to increased patient comorbidities and age, rapidly evolving changes in care standards, and the emergence of complex genome-guided, personalized therapies [12,13]. As a result of increasing patient complexity, current evidence and practice guidelines may not be directly applicable to many patients [12]. Physicians cite expert authorities as the best source for questions on complex clinical situations [6,7]. Questions to experts tend to be about nuanced patient situations and often require guidance, affirmation, judgment, and feedback [14]. However, expert insights are often not readily accessible to community physicians who treat 80% of patients with cancer in the United States [15]. Additionally, while there is a systematic process of documenting the best research evidence in journals and textbooks, there exists no centralized way of documenting and disseminating clinical expertise; it is found in conversations in conferences, hospital hallways, emails, and on the telephone. This valuable experiential knowledge is shared socially among colleagues, but it never makes its way to the greater community through an indexed and searchable database.

In most cancer centers, difficult clinical questions are discussed at multidisciplinary TBs in which peer expertise is shared. TBs are the central forums for decision-making in situations where there is limited evidence and in which patients have confounding factors. At the NCI-CCC, the TBs are more than a place for decision-making. They are acknowledged as a place for disseminating knowledge [16], generating ideas that lead to research projects, raising awareness of clinical trials [17], highlighting nuances in diagnostic approaches and treatment, educating oncology teams, and discussing existing controversies in treatments [8].

TheMednet has emerged as a novel physician-only website that has given oncologists a platform to ask and search answers to complex clinical questions. In collaboration with the TBs at the NCI-CCC, academics have shared knowledge outside the walls of 23 academic programs with oncologists across the United States. Over the course of 4+ years, discussions from these TBs have reached over 7000 oncologists across all 50 states of the United States. It is exemplary of how technology is helping to break down health disparities and achieve health equity. The discussions happening at major academic institutions have helped oncologists treat patients in rural settings, without the need for those patients to travel across the country. The observation that more than three quarters of these Q&As were viewed every month indicates that these clinically relevant Q&As provide value for weeks, months, and even years after they are posted. Answers are frequently updated as practices evolve, and new data become available. Our data indicate a high level of engagement from community oncologists and a high retention rate among the participating NCI-CCCs, with direct feedback from academic site leaders indicating high satisfaction with continued learning and professional growth. Additionally, when asked about the impact of the TB Q&As, 1 in 5 responses indicate that these Q&As may change oncologists’ future practice.

This program has been unique as it involves active participation and interaction of academic and community oncologists. Additionally, the answers display how academic physicians incorporate current guidelines and evidence into their clinical practice based on their years of experience and research in the field. This access to up-to-date expert knowledge helps community oncologists with clinical decision-making by affirming their current practices, teaching them new information they did not previously know, and changing clinical practices. To our knowledge, this represents the only searchable repository of expert knowledge on areas of controversy in oncology, accessible to oncologists throughout the United States.

This program has gained unprecedented prominence and popularity in the 4 years since its launch, and engagement continues to increase over time. Future efforts will be focused on involving more NCI-CCCs in the program, expanding to additional disease sites such as pediatric and gynecologic oncology, in addition to malignant hematology, and international expansion to reach non–US-based physicians. Additionally, qualitative and quantitative studies will investigate how regular exposure to knowledge at the NCI-CCCs impacts patient care in community settings.

## Appendix

Multimedia Appendix 1 Responses to impact survey questions.

## Abbreviations

ASCO: American Society of Clinical Oncology
FDA: Food and Drug Administration
MDACC: MD Anderson Cancer Center
NCI-CCC: National Cancer Institute–designated Comprehensive Cancer Center
Q&A: question and answer
TB: tumor board

## References

[1] Siegel RL, Miller KD, Jemal A (2019). Cancer statistics, 2019. CA A Cancer J Clin.

[2] Oncology (cancer)/ hematologic malignancies approval notifications. FDA.

[3] New drug therapy approvals 2019. FDA.

[4] Del Fiol G, Workman TE, Gorman PN (2014). Clinical questions raised by clinicians at the point of care: a systematic review. JAMA Intern Med.

[5] Bennett N, Casebeer LL, Zheng S, Kristofco R (2006). Information-seeking behaviors and reflective practice. J Contin Educ Health Prof.

[6] Cook DA, Sorensen KJ, Wilkinson JM, Berger RA (2013). Barriers and decisions when answering clinical questions at the point of care: a grounded theory study. JAMA Intern Med.

[7] Ely J, Osheroff JA, Maviglia SM, Rosenbaum ME (2007). Patient-care questions that physicians are unable to answer. J Am Med Inform Assoc.

[8] Gatcliffe T, Coleman R (2008). Tumor board: more than treatment planning—a 1-year prospective survey. J Cancer Educ.

[9] Bristow RE, Chang J, Ziogas A, Campos B, Chavez LR, Anton-Culver H (2015). Impact of national cancer institute comprehensive cancer centers on ovarian cancer treatment and survival. J Am Coll Surg.

[10] Anderson AH (2012). Discovering value from community activity on focused question answering sites: a case study of stack overflow.

[11] Richardson M, Dominowska E, Robert R (2007). Predicting clicks: estimating the click-through rate for new ads.

[12] Sarfati D, Koczwara B, Jackson C (2016). The impact of comorbidity on cancer and its treatment. CA Cancer J Clin.

[13] Meric-Bernstam F, Johnson A, Holla V, Bailey AM, Brusco L, Chen K, Routbort M, Patel KP, Zeng J, Kopetz S, Davies MA, Piha-Paul SA, Hong DS, Eterovic AK, Tsimberidou AM, Broaddus R, Bernstam EV, Shaw KR, Mendelsohn J, Mills GB (2015). A decision support framework for genomically informed investigational cancer therapy. J Natl Cancer Inst.

[14] Smith R (1996). What clinical information do doctors need?. BMJ.

[15] Pfister DG, Rubin DM, Elkin EB, Neill US, Duck E, Radzyner M, Bach PB (2015). Risk adjusting survival outcomes in hospitals that treat patients with cancer without information on cancer Stage. JAMA Oncol.

[16] Siderits R, Yates S, Rodriguez A, Lee T, Rimmer C, Roche M (2011). Embedding QR codes in tumor board presentations, enhancing educational content for oncology information management. J Registry Manag.

[17] Coe K, Wilson C, Eisenberg M, Attakai A, Lobell M (2006). Creating the environment for a successful community partnership. Cancer.

