# Supplementary figures and images for: Making National Cancer Institute–Designated Comprehensive Cancer Center Knowledge Accessible to Community Oncologists via an Online Tumor Board: Longitudinal Observational Study
Source: JMIR Cancer. 2022 May 19;8(2):e33859. doi: 10.2196/33859 (PMC9164098; doi:10.2196/33859)

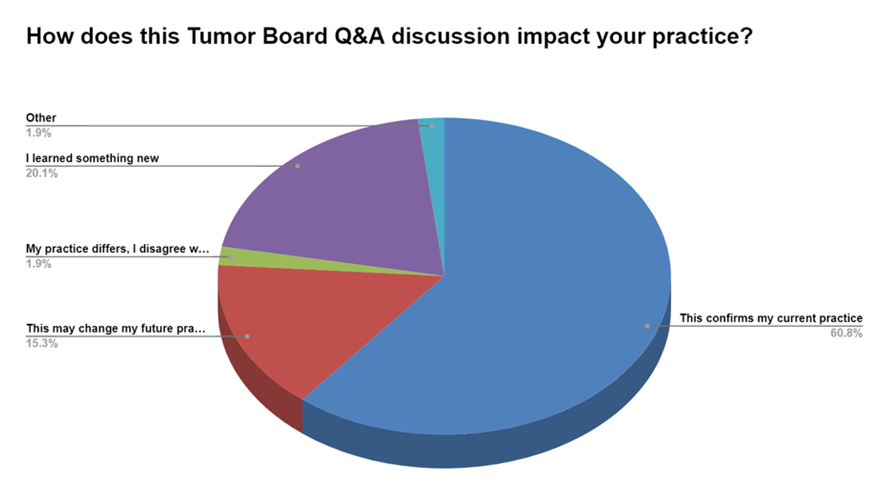

Supplement: Multimedia Appendix 1 [file cancer_v8i2e33859_app1.png]
